# Categorical Properties of Soft Sets

**DOI:** 10.1155/2014/783056

**Published:** 2014-08-18

**Authors:** Min Zhou, Shenggang Li, Muhammad Akram

**Affiliations:** ^1^College of Mathematics and Information Sciences, Shaanxi Normal University, Xi'an 710119, China; ^2^Department of Mathematics, University of the Punjab, New Campus, Lahore, Pakistan

## Abstract

The present study investigates some novel categorical properties of soft sets. By combining categorical theory with soft set theory, a categorical framework of soft set theory is established. It is proved that the category **SFun** of soft sets and soft functions has equalizers, finite products, pullbacks, and exponential properties. It is worth mentioning that we find that **SFun** is both a topological construct and Cartesian closed. The category **SRel** of soft sets and *Z*-soft set relations is also characterized, which shows the existence of the zero objects, biproducts, additive identities, injective objects, projective objects, injective hulls, and projective covers. Finally, by constructing proper adjoint situations, some intrinsic connections between **SFun** and **SRel** are established.

## 1. Introduction

It is well known that many traditional mathematical tools such as fuzzy set theory, probability theory, rough set theory, and interval mathematic theory have their own limitations in dealing with some uncertain problems caused by the incompatibility of various parameter tools. To overcome the difficulties mentioned above, Molodtsov [1] initiated soft set theory by introducing enough compatible parameters. In the context of soft set, researchers can choose freely the form of parameters to simplify the decision-making process, which often makes the process more efficient under the absence of partial information. Consequently, Ali et al. [2] further introduced some new operations in soft set theory. Recently, soft set theory has opened up keen insights and has a rich potential for application in many different fields such as ontology [3], data analysis [4, 5], forecasting [6], simulation [7], decision making [8–11], medical science [12], rule mining [13], algebraic systems [14–20], optimization [21], and textures classification [22]. However, being originated from relatively simple information models, classical soft set theory may not be suitable for those complex information models. In order to solve practical problems better by employing soft set theory, it is important to allure capable pure mathematicians to participate in the study of soft set theory. On the other hand, category theory is not only a basic tool for characterizing all kinds of mathematical structures, but also a tie which can connect easily the fields of mathematics and theoretical computer science (see [23–28]). Many researchers (see [29]) even argue that it is category theory, rather than set theory, that provides the proper setting for the study of pure mathematics. Based on the above analysis, a natural question is whether we can research by combining soft set theory with category theory. The fact is that there exists some categorical concepts, such as product, in soft set theory. Moreover, category theory has been successfully applied to fuzzy set theory [30, 31] and rough set theory [32, 33]. In 2007, Aktaş and Çağman [15] showed that both a fuzzy set and a rough set can be regarded as a soft set, which makes it possible to investigate soft set theory and category theory in a common setting. Inspired by this, recently, Zahiri [34] introduced a category whose objects are soft sets. Sardar and Gupta [35] defined another soft category which is a parameterized family of subcategories of a category. Varol et al. [36] defined a new category of soft sets and soft mapping. These studies have presented a preliminary, but potentially interesting, research direction. However, some basic problems still need further investigation. Based on these analyses, we further study the categorical framework of soft set theory in the present paper.

The main contributions of the paper have 3-fold. First, we show that the category** SFun** of soft sets and soft functions is Cartesian closed. On the one hand, because of the consistency of expression function between Cartesian closed category and *λ*-calculation with types, many researchers have been devoted to establishing all kinds of Cartesian closed categories in the universe theory for denotational semantics of computer programming language. On the other hand, soft set theory has been widely applied to many fields. Based on this, we further study the category** SFun** of soft sets and soft functions and prove that it is Cartesian closed. Second, we give a new characterization on soft set relations by employing category theory. There is no doubt that soft set relations play a significant role in the study of soft set theory and they can not only characterize the theoretical relations of two soft sets but also enrich the soft set theory. Presently, researches on soft set relations have received widespread attention and have made great progress (see [37–42]). Meanwhile, it is worth noting that category of binary relations has been widely applied to mathematics and computer science [43, 44]. Inspired by this, we make a further discussion on the category** SRel** of soft sets and *Z*-soft set relations. Third, we construct a concrete adjoint situation between the category** SFun** and** SRel** and characterize its basic relationships.

The remaining parts of the paper are arranged as follows.  Section 2 shows some preliminaries. We present in Section 3 the concept of soft functions and discuss the fundamental properties of the category** SFun**. In Section 4, the characterizations of the category** SRel** are investigated.  Section 5 focuses on studying the intrinsic connections between** SFun** and** SRel**.

## 2. Preliminaries

In this section, we recall some elementary notions and facts related to soft set theory [1], category theory (see [23–27]) which will be often used in this paper. In what follows, we denote by *U* an initial universe of objects and by *E* the set of parameters that relate to objects in *U*. *P*(*U*) presents the power set of *U*. *A*, *B*, *C*, and *J* are the subsets of *E*.

**Definition 1 (see [1]).**

A pair (*F*, *A*) is called a soft set over *U*, where *F* is a function given by *F* : *A* → *P*(*U*).

In other words, a soft set over *U* is a parameterized family of subsets of *U*. For any parameter *x* ∈ *A*, *F*(*x*) may be considered as the set of *x*-approximate elements of the soft set (*F*, *A*).

**Proposition 2 (see [24]).**

If a category *C* has finite products and equalizers, then *C* has pullbacks.

**Definition 3 (see [26]).**

Let *Z* be an object in a category *C*. One calls *Z* initial if for each object *A* there is exactly one morphism from *Z* to *A*; one calls *Z* terminal if for each object *A* there is exactly one morphism from *A* to *Z*; and one calls *Z* a zero object if it is both initial and terminal.

For objects *A*, *B* in a category with zero object *Z*, we use 0_*A*,*B*_ for the unique morphism *A* → *Z* → *B*.

**Definition 4 (see [26]).**

A category *C* with zero has biproducts if for each family {*A*_*i*_}_*i*∈*I*_ of objects there is an object ⊕_*I*_ *A*_*i*_, together with families of morphisms *μ*_*i*_ : *A*_*i*_ → ⊕_*I*_ *A*_*j*_ and *π*_*i*_ : ⊕_*I*_ *A*_*j*_ → *A*_*i*_ such that

- the morphisms *μ*_*i*_ : *A*_*i*_ → ⊕_*I*_ *A*_*j*_ are a coproduct of the family {*A*_*i*_}_*i*∈*I*_;
- the morphisms *π*_*i*_ : ⊕_*I*_ *A*_*j*_ → *A*_*i*_ are a product of the family {*A*_*i*_}_*i*∈*I*_;
- *π*_*i*_∘*μ*_*j*_ = *δ*_*ij*_ for each *i*, *j* ∈ *I*; here *δ*_*ij*_ is the identity map 1_*A*_*i*__ if *i* = *j* and the zero map 0_*A*_*i*_,*A*_*j*__ if *i* ≠ *j*.

**Example 5 (see [23]).**

In category** Rel** of sets and the relations between them, for a family of sets {*X*_*i*_}_*i*∈*I*_, let *X* be their disjoint union ⨆ _*I*_ *X*_*i*_ = {(*x*, *i*)∣*x* ∈ *X*_*i*_ for some *i* ∈ *I*} and define relations *μ*_*i*_ from *X*_*i*_ to *X* and *π*_*i*_ from *X* to *X*_*i*_ by setting *μ*_*i*_ = {(*x*, (*x*, *i*))∣*x* ∈ *X*_*i*_} and *π*_*i*_ = {((*x*, *i*), *x*)∣*x* ∈ *X*_*i*_}. Then the disjoint union *X* with morphisms *μ*_*i*_ and *π*_*i*_ is a biproduct of the family {*X*_*i*_}_*i*∈*I*_.

**Definition 6 (see [26]).**

A semiadditive category is a category *C* where each homset *C*(*B*, *C*) is equipped with the structure of a commutative monoid with operation + such that, for any *f* : *A* → *B*, *g*, *h* : *B* → *C*, and *k* : *C* → *D*,

(1) $$\begin{array}{c} \left(g+h\right)\circ f=\left(g\circ f\right)+\left(h\circ f\right),\\ k\circ \left(g+h\right)=\left(k\circ g\right)+\left(k\circ h\right). \end{array}$$

**Definition 7 (see [26]).**

An involution on a category *C* is a contravariant functor from *C* to itself of period two.

**Definition 8 (see [26]).**

Let *Z* be a *C*-object. Then *Z* is injective if, for every monic *f* : *X* → *Y* and each *g* : *X* → *Z*, there is an *h* : *Y* → *Z* with *g* = *h*∘*f*:

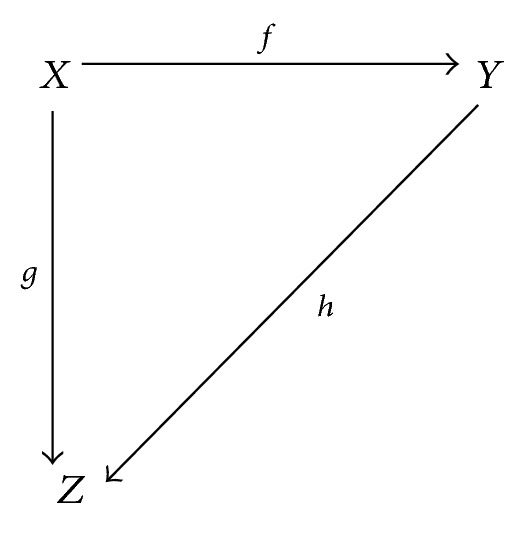
(2)
The map *e* : *X* → *Z* is called an injective hull of *X* if *e* is monic, *Z* is injective, and for any *k* : *Z* → *V* we have *k*∘*e* being monic which implies that *k* is monic.

**Definition 9 (see [27]).**

For categories *C* and *D* and functors *F* : *C* → *D* and *G* : *D* → *C*, one says (*F*, *G*) is an adjoint situation if *F* is left adjoint to *G* and *G* is right adjoint to *F*. This implies that, for objects *X* ∈ *C* and *Y* ∈ *D*, there is a natural isomorphism between the homsets *C*(*X*, *G*(*Y*)) ≈ *D*(*F*(*X*), *Y*).

**Definition 10 .**

One calls that a category *C* has exponential properties if it has finite products and for each of the *C*-objects *A*, *B*, there exists a *C*-object *B* ^*A*^ and a *C*-morphism *ev* : *B* ^*A*^ × *A* → *B* such that, for each *C*-object *D* and *C*-morphism *F* : *D* × *A* → *B*, there exists a unique *C*-morphism $\overset{-}{F}:D\to B^{A}$ with $ev\circ (\overset{-}{F}\times id_{A})=F$. That is, the diagram

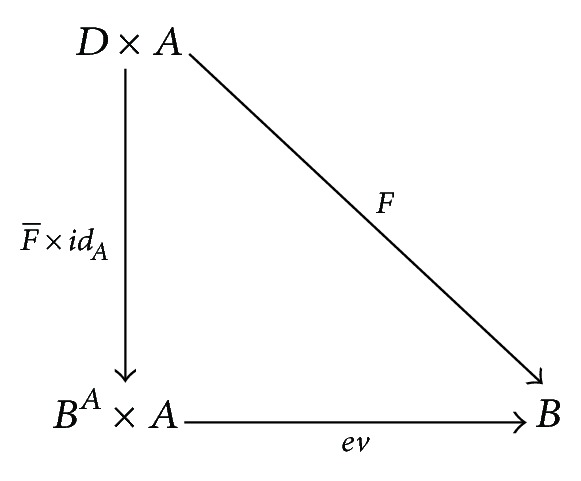
(3)

is commutative.

**Definition 11 (see [26]).**

A category *C* is called Cartesian closed if it has equalizers, finite products, terminal objects, and exponential properties.

For the other standard terminology of category theory, see [24, 26].

## 3. The Category SFun of Soft Sets and Soft Functions

The properties of the category** SFun** will be investigated in this section. Particularly, we will prove that** SFun** is a topological construct and Cartesian closed.

**Definition 12 .**

Let (*F*, *A*) and (*G*, *B*) be two soft sets over *U*. Then one says that the mapping *f* : *A* → *B* is a soft function from (*F*, *A*) to (*G*, *B*) if it satisfies *F*(*a*)⊆(*G*∘*f*)(*a*) for each *a* ∈ *A*.

**Example 13 .**

Let *U* = {*u*_1_, *u*_2_, *u*_3_, *u*_4_, *u*_5_, *u*_6_} be the set of candidate dresses and *E* = {*e*_1_, *e*_2_, *e*_3_, *e*_4_} the set of parameters, where *e*_*i*_ (*i* = 1,2, 3,4) stands for expensive, beautiful, elegant, and classical, respectively. Let *A* = {*e*_1_, *e*_2_}, *B* = {*e*_1_, *e*_4_}, *F*(*e*_1_) = {*u*_3_}, *F*(*e*_2_) = {*u*_1_, *u*_2_, *u*_6_}, *G*(*e*_1_) = {*u*_3_, *u*_5_}, and *G*(*e*_4_) = {*u*_1_, *u*_2_, *u*_5_, *u*_6_}. It is easy to check that (*F*, *A*) and (*G*, *B*) are two soft sets over *U*. Define a function *f* : *A* → *B* by *f*(*e*_1_) = *e*_1_, *f*(*e*_2_) = *e*_4_. By routine calculations, we can prove that *F*(*e*_1_)⊆(*G*∘*f*)(*e*_1_) and *F*(*e*_2_)⊆(*G*∘*f*)(*e*_2_). By Definition 12, *f* is a soft function from (*F*, *A*) to (*G*, *B*).

**Remark 14 .**

The concept of soft functions is different from soft set functions defined in [37].

Let** SFun** denote the category of all soft sets over *U* and soft functions. We next discuss the properties of the category** SFun**.

**Lemma 15 .**

**S** **F** **u** **n** has equalizers

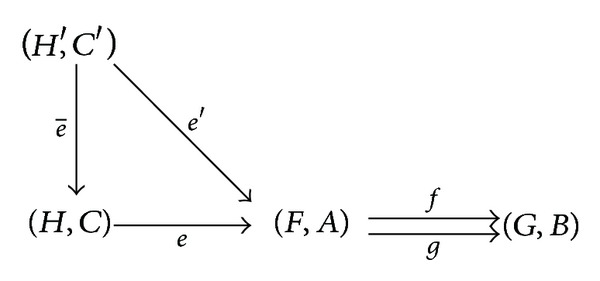
(4)

**Proof**

Suppose that (*F*, *A*) and (*G*, *B*) are two** SFun**-objects over *U*; *f* and *g* are two** SFun**-morphisms from (*F*, *A*) to (*G*, *B*). Define *C* = {*a* ∈ *A* : *f*(*a*) = *g*(*a*)}, *e* : *C* → *A*, an embedding, and *H* = *F*∘*e*. From the assumption, we can easily know that (*H*, *C*) is a** SFun**-object, *f*∘*e* = *g*∘*e*, and *H*(*c*) = (*F*∘*e*)(*c*) for each *c* ∈ *C*. Thus *e* is a** SFun**-morphism. We next show that ((*H*, *C*), *e*) is the equalizer of *f* and *g*. Assume that (*H*′, *C*′) is a** SFun**-object and *e*′ is a** SFun**-morphism from (*H*′, *C*′) to (*F*, *A*) satisfying *f*∘*e*′ = *g*∘*e*′. Define a mapping $\overset{-}{e}:C^{\prime }\to C$ and $\overset{-}{e}=e^{\prime }$. In what follows we focus on showing that $\overset{-}{e}$ is a** SFun**-morphism from (*H*′, *C*′) to (*H*, *C*) and $e^{\prime }=e\circ \overset{-}{e}$. Firstly, by *f*∘*e*′ = *g*∘*e*′, we can infer that *f*(*e*′(*c*′)) = *g*(*e*′(*c*′)) for each *c*′ ∈ *C*′, which means that *e*′(*c*′) ∈ *C*. Hence $\overset{-}{e}=e^{\prime }$ is well defined. Secondly, according to $H=F\circ e,\overset{-}{e}=e^{\prime }$, and *e*′ being a** SFun**-morphism, we have

(5) H′(c′)⊆F(e′(c′))=F(e−(c′))=F(e(e−(c′)))=(F∘e)(e−(c′))=H(e−(c′)),

where *c*′ ∈ *C*′. Therefore, $\overset{-}{e}$ is a** SFun**-morphism. At last, from the assumption, we know that $e^{\prime }=e\circ \overset{-}{e}$ and $\overset{-}{e}$ is unique. In conclusion, ((*H*, *C*), *e*) is the equalizer of *f* and *g*.

**Lemma 16 .**

**S** **F** **u** **n** has finite products

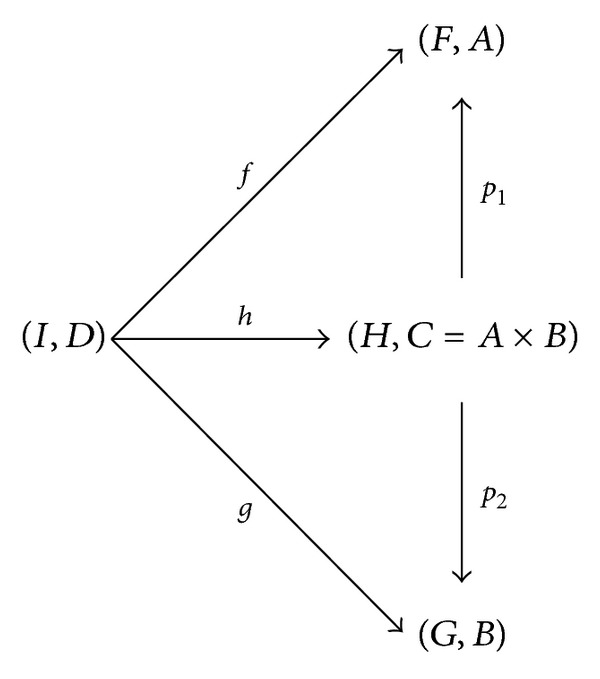
(6)

**Proof**

Firstly, let (*F*, *A*) and (*G*, *B*) be two** SFun**-objects. Define three mappings

(7) H:A×B⟶P(U) (a,b)⟶F(a)∩G(b),p1:A×B⟶A (a,b)⟶a,p2:A×B⟶B (a,b)⟶b,

whence, for each (*a*, *b*) ∈ *A* × *B*,

(8) $$\begin{array}{c} H\left(a,b\right)=F\left(a\right)\cap G\left(b\right)\subseteq F\left(a\right)=F\left(P_{1}\left(a,b\right)\right). \end{array}$$

It follows that *p*_1_ is a** SFun**-morphism. By the same argument, *p*_2_ is also a** SFun**-morphism. Secondly, for each** SFun**-object (*I*, *D*), suppose that *f* and *g* are** SFun**-morphisms from (*I*, *D*) to (*F*, *A*) and (*G*, *B*), respectively. Then *I*(*d*)⊆*F*(*f*(*d*)) and *I*(*d*)⊆*G*(*g*(*d*)) for every *d* ∈ *D*. Further, define a mapping

(9) h:D⟶A×B d⟶(f(d),g(d)).

Then we can infer that, for each *d* ∈ *D*,

(10) I(d)⊆F(f(d))∩G(g(d))=H(f(d),g(d))=H(h(d))=(H∘h)(d),

which yields that *h* is a** SFun**-morphism. At last, for every *d* ∈ *D*, one obtains

(11) $$\begin{array}{c} \left(p_{1}\circ h\right)\left(d\right)=p_{1}\left(h\left(d\right)\right)=p_{1}\left(f\left(d\right),g\left(d\right)\right)=f\left(d\right). \end{array}$$

Therefore, *p*_1_∘*h* = *f*. Analogously, *p*_2_∘*h* = *g*. Apparently, *h* is unique. In conclusion, {(*H*, *C*), *p*_1_, *p*_2_} is a finite product of (*F*, *A*) and (*G*, *B*).

**Theorem 17 .**

**SFun** has pullbacks.

**Proof**

By Proposition 2, it is a direct consequence of Lemmas 15 and 16.

**Lemma 18 .**

**S** **F** **u** **n** has terminal objects.

**Proof**

Define a mapping

(12) T{∅}:{∅}⟶P(U) ∅⟶U.

Trivially, (*T*_{*∅*}_, {*∅*}) is a** SFun**-object. For every** SFun**-object (*T*_*M*_, *M*), define a mapping

(13) $$\begin{array}{c} f:M⟶\left\{\emptyset \right\}\;\;\text{by}\;\;m⟶\emptyset . \end{array}$$

Then for each *m* ∈ *M*, it holds that

(14) $$\begin{array}{c} T_{M}\left(m\right)\subseteq T_{\left\{\emptyset \right\}}\left(f\left(m\right)\right)=T_{\left\{\emptyset \right\}}\left(\emptyset \right)=U, \end{array}$$

which implies that *f* is a** SFun**-morphism from (*T*_*M*_, *M*) to (*T*_{*∅*}_, {*∅*}). It is easy to know that *f* is unique. By Definition 3, (*T*_{*∅*}_, {*∅*}) is a terminal object of** SFun**.

**Proposition 19 .**

**S** **F** **u** **n** has initial objects.

**Proof**

The proof runs parallel to that of Lemma 18.

According to Definition 3, we can easily obtain the following proposition.

**Proposition 20 .**

**S** **F** **u** **n** has zero objects.

**Lemma 21 .**

**S** **F** **u** **n** has exponential properties.

**Proof**

Assume that (*F*, *A*) and (*G*, *B*) are two** SFun**-objects over *U*; *B* ^*A*^ = {*f*∣*f* : *A* → *B* is a mapping}. For all *f* ∈ *B* ^*A*^, define

(15) αf={t∈P(U) ∣ F(a)∩t⊆G(f(a)),∀a∈A},H1(f)=∪{t ∣ t∈αf},C=supp⁡H1={f ∣ H1(f)≠∅},H(f)=H1(f).

Then *C* ≠ *∅*. In fact, choose *b* ∈ *B* and define a mapping *f*′ : *A* → *B* by *a* → *b* such that *t* = *G*(*b*) ≠ *∅*. whence *t* ∈ *α*_*f*_′, which meas that *f*′ ∈ *C*. That is, *C* ≠ *∅*.

From the definitions, we can easily know that (*H*, *C*) is a** SFun**-object. Define the evaluation mapping as follows:

(16) ev:(H,C)×(F,A)⟶(G,B)

(17) $$\begin{array}{c} \left(f,a\right)⟶f\left(a\right). \end{array}$$

Then *t*∩*F*(*a*)⊆*G*(*f*(*a*)) for all *t* ∈ *α*_*f*_. It is immediate that

(18) $$\begin{array}{c} G\left(f\left(a\right)\right)⊇\left(\cup \left\{t\;|\;t\in \alpha_{f}\right\}\right)\cap F\left(a\right)=H\left(f\right)\cap F\left(a\right), \end{array}$$

which implies that *ev* is a** SFun**-morphism. Furthermore, we show that *ev* has the couniversal property. Assume that (*Z*, *J*) is a** SFun**-object such that *Z*(*j*) ≠ *∅* for every *j* ∈ *J* and *g* : (*Z*, *J*)×(*F*, *A*)→(*G*, *B*) is a** SFun**-morphism. It remains to prove that there exists a unique** SFun**-morphism $\overset{-}{g}:(Z,J)\to (H,C)$ such that $ev\circ (\overset{-}{g}\times id_{A})=g$. Firstly, for every *j* ∈ *J*, define

(19) g−(j):A⟶B   a⟶g−(j)(a)=g(j,a).

Since *g* is a** SFun**-morphism, one has

(20) $$\begin{array}{c} Z\left(j\right)\cap F\left(a\right)\subseteq G\left(g\left(j,a\right)\right)\;\text{for}\;\text{each}\;\;j\in J,a\in A. \end{array}$$

Consequently,

(21) $$\begin{array}{c} Z\left(j\right)\cap F\left(a\right)\subseteq G\left(\overset{-}{g}\left(j\right)\left(a\right)\right)\;\text{for}\;\text{each}\;\;j\in J,a\in A, \end{array}$$

which implies that $Z(j)\in \alpha_{\overset{-}{g}(j)}$. Further, according to the assumption, $H(\overset{-}{g}(j))⊇Z(j)\neq \emptyset$. Hence $\overset{-}{g}(j)\in C$ and $\overset{-}{g}$ is a** SFun**-morphism from (*Z*, *J*) to (*H*, *C*). Secondly, for each (*j*, *a*) ∈ *J* × *A*, it holds that

(22) (ev∘(g−×idA))(j,a)=ev((g−×idA)(j,a))=ev(g−(j),a)=g−(j)(a)=g(j,a),

whence $ev\circ (\overset{-}{g}\times id_{A})=g$. Namely, the diagram

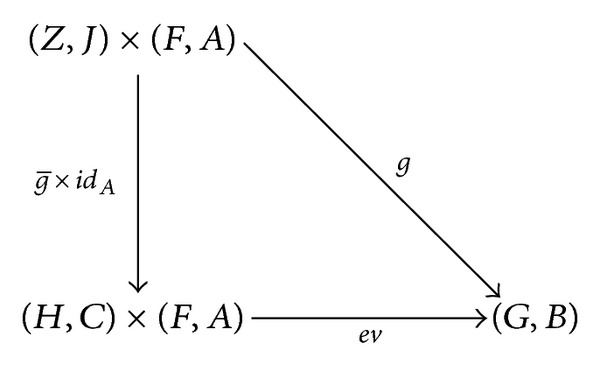
(23)

is commutative. Furthermore, suppose that *g*′ : (*Z*, *J*)→(*H*, *C*) is a** SFun**-morphism satisfying *ev*∘(*g*′ × *id*_*A*_) = *g*. Then for every *j* ∈ *J* and *a* ∈ *A*,

(24) $$\begin{array}{c} g\left(j,a\right)=ev\circ \left(g^{\prime }\times id_{A}\right)\left(j,a\right)=ev\left(g^{\prime }\left(j\right),a\right)=g^{\prime }\left(j\right)\left(a\right). \end{array}$$

On the other hand, we have

(25) $$\begin{array}{c} g\left(j,a\right)=ev\circ \left(\overset{-}{g}\times id_{A}\right)\left(j,a\right)=ev\left(\overset{-}{g}\left(j\right),a\right)=\overset{-}{g}\left(j\right)\left(a\right). \end{array}$$

Thus $\overset{-}{g}(j)(a)=g^{\prime }(j)(a)$. Since *j* and *a* are arbitrary, $\overset{-}{g}=g^{\prime }$. This completes the proof.

Now we are ready to present two of our main results as follows.

**Theorem 22 .**

**S** **F** **u** **n** is Cartesian closed.

**Proof**

Lemmas 15, 16, 18, and 21 prove the claim.

**Theorem 23 .**

**S** **F** **u** **n** is a topological construct.

**Proof**

Let {(*F*_*i*_, *A*_*i*_)}_*i*∈*I*_ be a family of** SFun**-objects indexed by a class *I* and {*f*_*i*_∣*A*→*A*_*i*_}_*i*∈*I*_ a family of mappings. Define a soft set over *U* as follows:

(26) F:A⟶P(U) a⟶⋂i∈I(Fi(fi(a))).

Then (*F*, *A*) ∈ *O*b(**SFun**). It suffices to show that {*f*_*i*_ : (*F*, *A*)→(*F*_*i*_, *A*_*i*_)}_*i*∈*I*_ is the unique** SFun** initial lift of {*f*_*i*_ : *A* → *A*_*i*_}_*i*∈*I*_. Next, we complete the proof by the following two steps.

*Step 1*. We show that {*f*_*i*_ : (*F*, *A*)→(*F*_*i*_, *A*_*i*_)}_*i*∈*I*_ is a** SFun** initial lift of {*f*_*i*_ : *A* → *A*_*i*_}_*i*∈*I*_. Firstly, we claim that *f*_*i*_ : (*F*, *A*)→(*F*_*i*_, *A*_*i*_) is a family of** SFun**-morphisms for every *i* ∈ *I*. By the assumption, for each *a* ∈ *A* and *i* ∈ *I*, one yields

(27) $$\begin{array}{c} F\left(a\right)=\underset{i\in I}{⋂}\left(F_{i}\left(f_{i}\left(a\right)\right)\right)\subseteq F_{i}\left(f_{i}\left(a\right)\right), \end{array}$$

whence {*f*_*i*_}_*i*∈*I*_ is a family of** SFun**-morphisms. Furthermore, suppose that (*G*, *B*) ∈ *O*b(**SFun**), *g* : *B* → *A* is a mapping such that *g*_*i*_ = *f*_*i*_∘*g* for every *i* ∈ *I*, and *g*_*i*_ : (*G*, *B*)→(*F*_*i*_, *A*_*i*_) is a family of** SFun**-morphisms. Then, we can infer that *G*(*b*)⊆*F*_*i*_(*g*_*i*_(*b*)) for all *i* ∈ *I* and *b* ∈ *B*. It follows that

(28) G(b)⊆⋂i∈IFi(gi(b))=⋂i∈I(Fi(fi∘g)(b))=⋂i∈I(Fi(fi(g(b))))=F(g(b)).

Therefore, *g* is a** SFun**-morphism from (*G*, *B*) to (*F*, *A*). By definition, we can know that {*f*_*i*_ : (*F*, *A*)→(*F*_*i*_, *A*_*i*_)}_*i*∈*I*_ is a** SFun** initial lift of {*f*_*i*_ : *A* → *A*_*i*_}_*i*∈*I*_.

*Step 2*. We show the uniqueness of the initial lift. If ${\left\{f_{i}:(\overset{-}{F},A)\to (F_{i},A_{i})\right\}}_{i\in I}$ is also a** SFun** initial lift of {*f*_*i*_ : *A* → *A*_*i*_}_*i*∈*I*_ which is different from {*f*_*i*_ : (*F*, *A*)→(*F*_*i*_, *A*_*i*_)}_*i*∈*I*_, then ${\left\{f_{i}:(\overset{-}{F},A)\to (F_{i},A_{i})\right\}}_{i\in I}$ is a family of** SFun**-morphisms. It is immediate that $\overset{-}{F}(a)\subseteq F_{i}(f_{i}(a))$ for each *i* ∈ *I* and *a* ∈ *A*. Consequently, $\overset{-}{F}(a)\subseteq {⋂}_{i\in I}^{}F_{i}(f_{i}(a))=F(a)$. That is, $\overset{-}{F}\subseteq F$. On the other hand, for the** SFun**-object (*F*, *A*) and identity mapping *Id*_*A*_ : *A* → *A*, since ${\left\{f_{i}:(\overset{-}{F},A)\to (F_{i},A_{i})\right\}}_{i\in I}$ is a** SFun** initial lift of {*f*_*i*_ : *A* → *A*_*i*_}_*i*∈*I*_, we have *f*_*i*_∘*id*_*A*_ = *f*_*i*_, *f*_*i*_ is a family of** SFun**-morphisms for all *i* ∈ *I*, and $id_{A}:(F,A)\to (\overset{-}{F},A)$ is also a** SFun**-morphism. Therefore, $F(a)\subseteq \overset{-}{F}(id_{A}(a))=\overset{-}{F}(a)$ for each *a* ∈ *A*, which means that $F\subseteq \overset{-}{F}$. To sum up, $F=\overset{-}{F}$. Based on Steps 1 and 2,** SFun** is a topological construct.

## 4. The Category SRel of Soft Sets and *Z*-Soft Set Relations

The main aim of this section is to investigate the properties of the category** SRel**. We will begin with the analysis of the existence of the zero object, biproduct, and additive identity of** SRel**. Then the injective object, projective object, injective hull, and projective cover of** SRel** will be studied.

**Definition 24 (see [37]).**

Let (*F*, *A*) and (*G*, *B*) be two soft sets over *U*. Then the Cartesian product of (*F*, *A*) and (*G*, *B*) is defined as (*F*, *A*)×(*G*, *B*) = (*H*, *A* × *B*), where *H* : *A* × *B* → *P*(*U* × *U*) and *H*(*a*, *b*) = *F*(*a*) × *G*(*b*) for all (*a*, *b*) ∈ *A* × *B*; that is, *H*(*a*, *b*) = {(*h*_*i*_, *h*_*j*_)∣*h*_*i*_ ∈ *F*(*a*) and *h*_*j*_ ∈ *G*(*b*)}.

**Definition 25 (see [37]).**

Let (*F*, *A*) and (*G*, *B*) be two soft sets over *U*. Then a relation from (*F*, *A*) to (*G*, *B*) is a soft subset of (*F*, *A*)×(*G*, *B*).

In other words, a relation from (*F*, *A*) to (*G*, *B*) is of the form (*H*_1_, *S*), where *S* ⊂ *A* × *B* and *H*_1_(*a*, *b*) = *H*(*a*, *b*), for every (*a*, *b*) ∈ *S*; here (*H*, *A* × *B*) = (*F*, *A*)×(*G*, *B*) has been defined in Definition 24. Any subset of (*F*, *A*)×(*F*, *A*) is called a relation on (*F*, *A*).

From Definition 25, we can see that the condition for soft set relation between two soft sets is very weak but just the weak conditions of the soft set relation make those many elements satisfying the above definition in fact unrelated in actual problems. It can be illustrated clearly by the following example.

**Example 26 .**

Consider the soft set (*F*, *A*) which describes “the cost of the mobile phones” and the soft set (*G*, *B*) which describes the “attractiveness of mobile phones.” Assume that *U* = {*m*_1_, *m*_2_, *m*_3_, *m*_4_, *m*_5_, *m*_6_} is the universe consisting of six mobile phones, and the parameter sets is given by *A* = {*e*_1_, *e*_2_, *e*_3_} and *B* = {*e*_1_, *e*_4_, *e*_5_}, respectively, where *e*_*i*_ (*i* = 1,2, 3,4, 5) stands for “very cheap,” “costly,” “very costly,” “beautiful,” and “accessible,” respectively. Let *F*(*e*_1_) = {*m*_1_, *m*_3_}, *F*(*e*_2_) = {*m*_1_, *m*_4_}, *F*(*e*_3_) = {*m*_1_, *m*_5_}, *G*(*e*_1_) = {*m*_1_, *m*_3_, *m*_5_}, *G*(*e*_4_) = {*m*_1_, *m*_2_, *m*_3_, *m*_4_, *m*_6_}, and *G*(*e*_5_) = {*m*_5_}. Let (*F*, *A*)×(*G*, *B*) = (*H*, *A* × *B*). The relation *R* from (*F*, *A*) to (*G*, *B*) is given by (*H*_1_, *S*), where *S* = {(*e*_1_, *e*_1_), (*e*_1_, *e*_5_)}⊆*A* × *B*, *H*_1_(*a*, *b*) = *H*(*a*, *b*) for every (*a*, *b*) ∈ *S*. By Definition 25, *R* = {*F*(*e*_1_) × *G*(*e*_1_), *F*(*e*_1_) × *G*(*e*_5_)}.

In the above example, *F*(*e*_1_) = {*m*_2_, *m*_3_}, *G*(*e*_5_) = {*m*_5_}. It is obvious that there is no relation between them. However, *F*(*e*_1_) × *G*(*e*_5_) ∈ *R*, whence Definition 25 cannot describe precisely the relation between soft sets. To overcome this limitation, we strengthen the concept of soft set relations by defining a new soft set relation.

**Definition 27 .**

Let (*F*, *A*) and (*G*, *B*) be two soft sets over *U*. Then a *Z*-soft set relation *R* from (*F*, *A*) to (*G*, *B*) is a subset of *A* × *B* defined as

(29) $$\begin{array}{c} \left(a,b\right)\in R⟺F\left(a\right)\subseteq G\left(b\right),\;\text{where}\;\;a\in A,b\in B. \end{array}$$

The definition can be illustrated by diagrams of the form

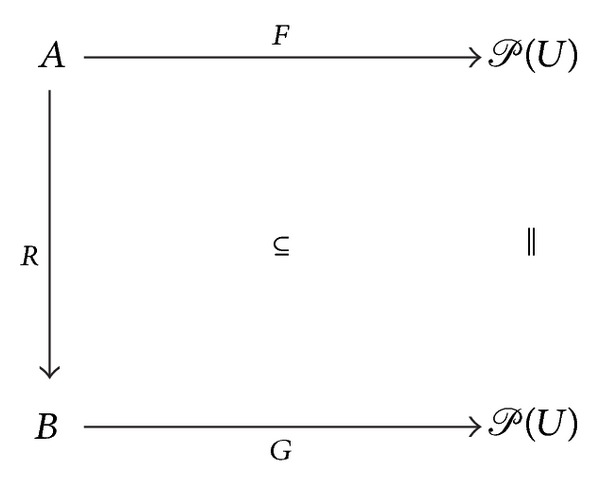
(30)

**Example 28 .**

Let (*F*, *A*) and (*G*, *B*) be the soft sets defined in Example 26. By Definition 27, we have *R* = {(*e*_1_, *e*_1_), (*e*_1_, *e*_4_), (*e*_2_, *e*_4_), (*e*_3_, *e*_1_)}.

**Definition 29 .**

Let *R* be a *Z*-soft set relation from (*F*, *A*) to (*G*, *B*) and *S* a *Z*-soft set relation from (*G*, *B*) to (*H*, *C*). Then the composition of *R* and *S*, denoted by *S*∘*R*, is defined as follows:

(31) S∘R={(a,c) ∣ ∀a∈A,∀c∈C,   there  is  a  b  in  B  with  aRb  and  bSc}.

**Definition 30 .**

Let (*F*, *A*) be a soft set over *U*. The identity *Z*-soft set relation *I*_*A*_ on (*F*, *A*) is defined as *I*_*A*_ = {(*a*, *a*)∣*a* ∈ *A*}.

**Proposition 31 .**

Let *R* be a *Z*-soft set relation from (*F*, *A*) to (*G*, *B*), *S* a *Z*-soft set relation from (*G*, *B*) to (*F*, *A*), and *I*_*A*_ an identity *Z*-soft set relation on (*F*, *A*). Then *R*∘*I*_*A*_ = *R* and *I*_*A*_∘*S* = *S*.

**Remark 32 .**

From the aforementioned definitions and propositions, we can construct a category, denoted by** SRel**, whose objects are all soft sets and morphisms are all *Z*-soft set relations.

**Proposition 33 .**

The category **S** **F** **u** **n** of soft sets and soft functions is the subcategory of **S** **R** **e** **l**.

**Proposition 34 .**

The empty set (with the empty function into *P*(*U*)) is a zero object in **S** **R** **e** **l**.

**Proof**

For each** SRel**-object (*F*, *A*), there exist unique morphisms

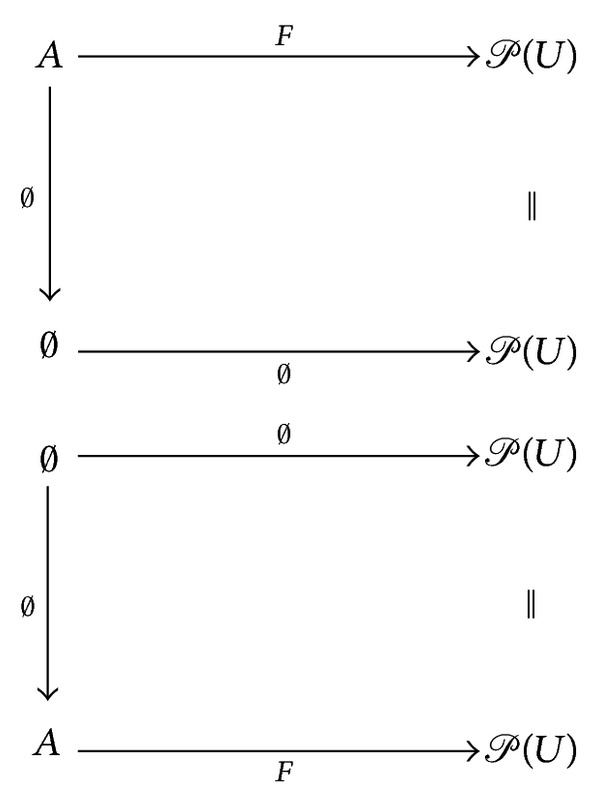
(32)

The inequalities are satisfied by default, whence the sets **S** **R** **e** **l**((*F*, *A*), (*∅*, *∅*)) and **S** **R** **e** **l**((*∅*, *∅*), (*F*, *A*)) each contain exactly one morphism.

**Proposition 35 .**

**S** **R** **e** **l** has biproducts.

**Proof**

Let {(*F*_*i*_, *A*_*i*_)}_*i*∈*I*_ be a family of** SRel**-objects and *A* = ⨆ _*I*_ *A*_*i*_ = {(*a*_*i*_, *i*)∣*a*_*i*_ ∈ *A*_*i*_ for some *i* ∈ *I*} the disjoint union of *A*_*i*_. Define a mapping *F* : *A* → *P*(*U*) as *F*(*a*_*i*_, *i*) = *F*_*i*_(*a*_*i*_). Further, define relations *q*_*i*_ from *A*_*i*_ to *A* and *p*_*i*_ from *A* to *A*_*i*_ as follows:

(33) $$\begin{array}{c} q_{i}=\left\{\left(a_{i},\left(a_{i},i\right)\right)\;|\;a_{i}\in A_{i}\right\},\;\;p_{i}=\left\{\left(\left(a_{i},i\right),a_{i}\right)\;|\;a_{i}\in A_{i}\right\}. \end{array}$$

We next show that (*F*, *A*) with morphisms *p*_*i*_ and *q*_*i*_ is a biproduct of the family {(*F*_*i*_, *A*_*i*_)}_*i*∈*I*_ by the following four steps.

*Step 1*. We first prove that *p*_*i*_ and *q*_*i*_ are** SRel**-morphisms. In fact, take an element (*a*_*i*_, (*a*_*i*_, *i*)) in *q*_*i*_; since *F*_*i*_(*a*_*i*_) = *F*(*a*_*i*_, *i*) for each *i* ∈ *I* and *a*_*i*_ ∈ *A*_*i*_, we have *F*_*i*_(*a*_*i*_)⊆*F*(*a*_*i*_, *i*), which means that *q*_*i*_ is a *Z*-soft set relation from (*F*_*i*_, *A*_*i*_) to (*F*, *A*). That is, *q*_*i*_ is a** SRel**-morphism from (*F*_*i*_, *A*_*i*_) to (*F*, *A*) for each *i* ∈ *I*. Analogously, we can prove that *p*_*i*_ is a** SRel**-morphism from (*F*, *A*) to (*F*_*i*_, *A*_*i*_) for every *i* ∈ *I*.

*Step 2*. We show that *q*_*i*_ are the morphisms for a coproduct. Suppose that (*G*, *B*) is a** SRel**-object and *R*_*i*_ : (*F*_*i*_, *A*_*i*_)→(*G*, *B*) is a family of** SRel**-morphisms. Define a relation *R* from *A* to *B* by (*a*_*i*_, *i*)*Rb* if and only if *a*_*i*_ *R*_*i*_ *b* for each *i* ∈ *I*. Firstly, we claim that *R* is a** SRel**-morphism from (*F*, *A*) to (*G*, *B*). If *a*_*i*_ *R*_*i*_ *b* for each *a*_*i*_ ∈ *A*_*i*_ and *b* ∈ *B*, since *R*_*i*_ : (*F*_*i*_, *A*_*i*_)→(*G*, *B*) is a family of** SRel**-morphisms for each *i* ∈ *I*, we have *F*_*i*_(*a*_*i*_)⊆*G*(*b*) for every *a*_*i*_ ∈ *A*_*i*_ and *b* ∈ *B*. On the other hand, *F*_*i*_(*a*_*i*_) = *F*(*a*_*i*_, *i*), which implies that *F*(*a*_*i*_, *i*)⊆*G*(*b*). By Definition 27, we have (*a*_*i*_, *i*)*Rb*, whence *R* is a** SRel**-morphism from (*F*, *A*) to (*G*, *B*). Secondly, we prove that *R*∘*q*_*i*_ = *R*_*i*_ for all *i* ∈ *I*. Let *a*_*i*_ ∈ *A*_*i*_ and *b* ∈ *B*; then by Definition 29, *a*_*i*_(*R*∘*q*_*i*_)*b* is equivalent to *a*_*i*_ *q*_*i*_(*a*_*i*_, *i*) and (*a*_*i*_, *i*)*Rb* for some (*a*_*i*_, *i*) ∈ *A*. According to assumption, (*a*_*i*_, *i*)*Rb* if and only if *a*_*i*_ *R*_*i*_ *b*, whence *R*∘*q*_*i*_ = *R*_*i*_. At last, the uniqueness is obvious. In conclusion, *q*_*i*_ are the morphisms for a coproduct.

*Step 3*. We further show that *p*_*i*_ are morphisms for a product. Assume that (*G*, *B*) is** SRel**-object and *S*_*i*_ : (*G*, *B*)→(*F*_*i*_, *A*_*i*_) is a family of** SRel**-morphisms. Define a relation *S* from *B* to *A* by setting *bS*(*a*_*i*_, *i*) if and only if *bS*_*i*_ *a*_*i*_. Similar to Step 2, we can infer that *S* is a unique morphism from (*G*, *B*) to (*F*, *A*) in** SRel** with *p*_*i*_∘*S* = *S*_*i*_. Thus *p*_*i*_ are morphisms for a product.

*Step 4*. Finally, a calculation shows that *p*_*i*_∘*q*_*i*_ is the identical relation on *A*_*i*_ if *i* = *j* and the empty relation from *A*_*j*_ to *A*_*i*_ if *i* ≠ *j*. Therefore, *p*_*i*_∘*q*_*j*_ = *δ*_*ij*_.

From the above discussion, we know that** SRel** has biproducts by Definition 4.

Any category with biproducts carries a unique semiadditive structure that can be defined via biproducts [26]. Next we briefly describe some properties of** SRel**.

**Proposition 36 .**

Let *R* and *S* be two **S** **R** **e** **l**-morphisms from (*F*, *A*) to (*G*, *B*). Then the semiadditive structure on homesets in **S** **R** **e** **l** is given by taking *R* + *S* to be the union of *Z*-soft set relations *R* ∪ *S*. In this case, the empty *Z*-soft set relation serves as the additive identity.

**Proof**

Let *R* and *S* be two** SRel**-morphisms from (*F*, *A*) to (*G*, *B*). Firstly, we show that *R* ∪ *S* is a** SRel**-morphism from (*F*, *A*) to (*G*, *B*). In fact, let *a* ∈ *A*, *b* ∈ *B*, and *a*(*R* ∪ *S*)*b*; then *a* *Rb* or *aSb*. In the first case, *R* is a** SRel**-morphism given by *F*(*a*)⊆*G*(*b*). And in the second case, *S* is a** SRel**-morphism given by *F*(*a*)⊆*G*(*b*), whence *R* ∪ *S* is a morphism in** SRel**. Secondly, we can easily verify that ∪ gives a commutative monoid structure on **S** **R** **e** **l**((*F*, *A*), (*G*, *B*)) with the empty *Z*-soft set relation as identity, and composition distributes over union.

**Proposition 37 .**

For each **S** **R** **e** **l**-object (*F*, *A*) and **S** **R** **e** **l**-morphism *R* : (*F*, *A*)→(*G*, *B*), there is an involution  ′ on **S** **R** **e** **l** defined as follows:

- (*F*,*A*)′ = (*F* ^*r*^, *A*), where *F* ^*r*^(*a*) = *U* − *F*(*a*) for all *a* ∈ *A*;
- *R*′ : (*G*,*B*)′ → (*F*,*A*)′ is the converse M-soft set relation *R* ^−1^, where *R* ^−1^ = {(*b*, *a*)∣*a* *Rb*, ∀*a* ∈ *A*, ∀*b* ∈ *B*}.

**Proof**

Let *a* ∈ *A*, *b* ∈ *B*, and *bR*′*a*. Since *R*′ is the converse *Z*-soft set relation of *R*, we have *a* *Rb*. In addition, *R* is a** SRel**-morphism, so *F*(*a*)⊆*G*(*b*) for every *a* ∈ *A* and *b* ∈ *B*, which means that *U* − *G*(*b*)⊆*U* − *F*(*a*). It is immediate that *R*′ is a** SRel**-morphism from (*G*,*B*)′ to (*F*,*A*)′. Furthermore, assume that *S* is a *Z*-soft set relation from (*G*, *B*) to (*H*, *C*); then by Definition 29, we can easily obtain that (*R*∘*S*)^−1^ = *S* ^−1^∘*R* ^−1^, whence  ′ is compatible with composition. At last, obviously,  ′ takes the identity map on (*F*, *A*) to the identity map on (*F*,*A*)′. Hence,  ′ is a contravariant functor that is obviously period two. By Definition 7,  ′ is an involution on** SRel**.

**Remark 38 .**

It should be noted that the notion of involution  ′ gives a bijective mapping from homset **S** **R** **e** **l**((*F*, *A*), (*G*, *B*)) to **S** **R** **e** **l**((*G* ^*r*^, *B*), (*F* ^*r*^, *A*)).

**Proposition 39 .**

Let *R* be a **S** **R** **e** **l**-morphism from (*F*, *A*) to (*G*, *B*). Then the following statements are equivalent:

- *R* is monic;
- if *C*⊆*A*, then the map *R*[·] : *P*(*A*) → *P*(*B*), defined by *R*[*C*] = {*b* ∈ *B*∣*c* *Rb* *for* *some* *c* ∈ *C*}, is one-one;
- for every *a* ∈ *A*, there exists *b* ∈ *B* such that *a* is the only element related to *b*.

**Proof**

(i)⇒(ii) Suppose that *C*, *D*⊆*A* and *R*[*C*] = *R*[*D*]. Take a singleton {∗} and assume that the map *∅* : {∗} → *P*(*U*) sends ∗ to *∅*. Define two relations *S*, *T* from {∗} to *A* by setting *S* = {(∗, *c*)∣*c* ∈ *C*} and *T* = {(∗, *d*)∣*d* ∈ *D*}. As *∅* is the subset of any sets, we have *S*, *T* : (*∅*, {∗})→(*F*, *A*) being** SRel**-morphisms and ∗*Sa*, ∗*Ta* for each *a* ∈ *A*. Since *R*[*C*] = *R*[*D*], by the definition, for each *b* ∈ *B*, there exist *c* ∈ *C* and *d* ∈ *D* such that *c* *Rb* if and only if *d* *Rb*. It is immediate that there exist *c* ∈ *C*⊆*A*, *d* ∈ *D*⊆*A* such that ∗*Sc* and *c* *Rb* if and only if ∗*Td* and *d* *Rb*. By Definition 29, *R*∘*S* = *R*∘*T*. Since *R* is monic, we have *S* = *T*. Consequently, *C* = *D*, which means that *R*[·] is one-one.

(ii)⇒(iii) Assume that *R*[·] : *P*(*A*) → *P*(*B*) is one-one; then *R*[*A* − {*a*}] ≠ *R*[*A*]. It follows from the definition that for each *a* ∈ *A* there exists *b* ∈ *B* such that *a* is the only element related to *b*.

(iii)⇒(i) Let *S*, *T* : (*H*, *C*)→(*F*, *A*) and *S* ≠ *T*; then we claim that there exist *c* ∈ *C* and *a* ∈ *A* such that (*c*, *a*) ∈ *S*, but (*c*, *a*) ∉ *T*. By (iii), take *b* ∈ *B* with *a*′*Rb*⇔*a*′ = *a*, and then it follows from Definition 29 that *c*(*R*∘*S*)*b*, but *c*(*R*∘*T*)*b* does not hold, which means that *R*∘*S* ≠ *R*∘*T*. Hence *R* is monic.

**Proposition 40 .**

Let *R* be a **S** **R** **e** **l**-morphism from (*F*, *A*) to (*G*, *B*). Then the following statements are equivalent:

- *R* is epic;
- the mapping *R* ^−1^ : *P*(*B*) → *P*(*A*), defined by *R* ^−1^[*D*] = {*a* ∈ *A*∣*aRd* *for* *some* *d* ∈ *D*}, is one-one;
- for every *b* ∈ *B*, there exists *a* ∈ *A* with *b* being the only element related to *a*.

**Proof**

The proof is similar to that of Proposition 39.

**Lemma 41 .**

Let *U* : *C* → *P*(*U*) be a map defined by *U*(*c*) = *U* for each *c* ∈ *C*. Then (*U*, *C*) is injective in **S** **R** **e** **l**.

**Proof**

For each** SRel**-objects (*F*, *A*), (*G*, *B*), let *R* : (*F*, *A*)→(*G*, *B*) be monic and *S* : (*F*, *A*)→(*U*, *C*). Assume that *B*_1_ = {*b* ∈ *B*∣ there  exists  exactly  one  a  such  that  *a* *Rb*}. Since *R* is monic, it follows from Proposition 39 that for every *a* ∈ *A* there exists *b* ∈ *B* such that *a* is the only element related to *b*. Define *T* : (*G*, *B*)→(*U*, *C*) by *T* = {(*b*, *c*)∣*b* ∈ *B*_1_  and  *a* *Sc*  for  some  *a* *Rb*}. Apparently, *T* is a** SRel**-morphism. According to the definition of *T*, we can infer that *T*∘*R* = *S*. That is, the following diagram

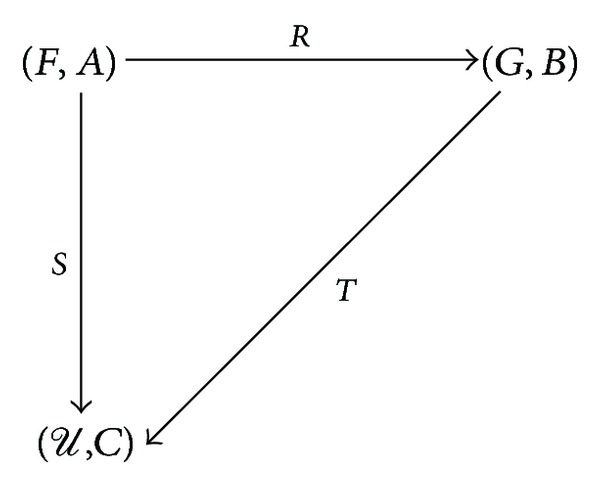
(34)

is commutative. By Definition 8, (*U*, *C*) is injective.

**Theorem 42 .**

Let (*F*, *A*) be a **S** **R** **e** **l**-object. Then the identity embedding *I* : (*F*, *A*)→(*U*, *A*) is an injective hull.

**Proof**

By Proposition 39, *I* is monic. In addition, according to Lemma 41, (*U*, *A*) is injective. Assume that *R* : (*U*, *A*)→(*G*, *B*) such that *R*∘*I* is monic. Because of a relation rather than a morphism, *R*∘*I* = *R*, whence Proposition 39 gives that *R* is monic. It follows from Definition 8 that *I* is an injective hull.

**Remark 43 .**

It is well known that projective objects of a category are dual to that of injective objects and projective covers are dual to that of injective hulls (see [26]). So we can easily obtain the following proposition.

**Theorem 44 .**

Let *∅* : *C* → *P*(*U*) be a mapping defined by *∅*(*c*) = *∅* for every *c* ∈ *C*. Then (*∅*, *C*) is the projective object of **S** **R** **e** **l**. Further, for each **S** **R** **e** **l**-object (*F*, *A*), the mapping *I* : (*∅*, *A*)→(*F*, *A*) is a projective cover of (*F*, *A*).

## 5. Adjoint Situations

We in this section mostly consider the relations among the categories** SFun**,** Set**,** SRel**, and** Rel**. In particular, we investigate the essential connections of** SFun** and** SRel** by means of adjoint situations.

**Definition 45 .**

Let *F*_1_ : **S** **F** **u** **n** → **S** **e** **t** be a forgetful functor which sends an object (*F*, *A*) to *A* and sends a morphism *R* : (*F*, *A*)→(*G*, *B*) to *R* : *A* → *B*.

Analogously, we can define another forgetful functor *G*_1_ : **S** **R** **e** **l** → **R** **e** **l**.

**Definition 46 .**

Define *F*_2_, *G*_2_ : **S** **e** **t** → **S** **F** **u** **n** and *F*_3_, *G*_3_ : **R** **e** **l** → **S** **R** **e** **l** for an object *A* and morphism *R* : *A* → *B* by setting

- *F*_2_(*A*) and *F*_3_(*A*) to be the object (*∅*, *A*), where *∅* : *A* → *P*(*U*) defined by *∅*(*a*) = *∅* for all *a* ∈ *A*;
- *G*_2_(*A*) and *G*_3_(*A*) to be the object (*U*, *A*), where *U* : *A* → *P*(*U*) defined by *U*(*a*) = *U* for all *a* ∈ *A*;
- *F*_2_(*R*), *F*_3_(*R*), *G*_2_(*R*), and *G*_3_(*R*) to be *R*, where *R* is considered with the appropriate domain and codomain.

**Theorem 47 .**

(i) The pair (*F*_3_, *G*_1_) is an adjoint situation.

(ii) The pair (*G*_1_, *G*_3_) is an adjoint situation.

(iii) The pair (*F*_2_, *F*_1_) is an adjoint situation.

(iv) The pair (*F*_1_, *G*_2_) is an adjoint situation.

**Proof**

We just prove (i) and (ii) because the proofs of (iii) and (iv) are similar.

(i) Let *A*, *B* be** Rel**-objects and (*G*, *B*)** SRel**-object. By Definition 46(i), *∅*(*a*) = *∅*⊆*G*(*b*) for all *a* ∈ *A* and *b* ∈ *B*; we can infer that a morphism *R* : *A* → *B* in** Rel** will lift to a morphism *R* : (*∅*, *A*)→(*G*, *B*) in** SRel**, whence **S** **R** **e** **l**((*∅*, *A*), (*G*, *B*)) ≈ **R** **e** **l**(*A*, *B*). According to Definitions 45 and 46(i), **S** **R** **e** **l**(*F*_3_(*A*), (*G*, *B*)) ≈ **R** **e** **l**(*A*, *G*_1_(*G*, *B*)). It follows from Definition 9 that (*F*_3_, *G*_1_) is an adjoint situation.

(ii) Assume that *A*, *B* are** Rel**-objects and (*G*, *B*) is a** SRel**-object. By Definition 46 (ii), *F*(*a*)⊆*U* = *U*(*b*) for every *a* ∈ *A* and *b* ∈ *B*, which means that a** Rel**-morphism *R* : *A* → *B* can lift to a** SRel**-morphism from (*F*, *A*) to (*U*, *B*). Thus **R** **e** **l**((*A*, *B*)) ≈ **S** **R** **e** **l**((*F*, *A*), (*U*, *B*)). By Definitions 45 and 46 (ii), **R** **e** **l**(*G*_1_(*F*, *A*), *B*) ≈ **S** **R** **e** **l**((*F*, *A*), *G*_3_(*B*)). Therefore, (*G*_1_, *G*_3_) is an adjoint situation according to Definition 9.

**Theorem 48 .**

Assume that *F*_4_ : **S** **F** **u** **n** → **S** **R** **e** **l** is the inclusion functor and define a functor

(35) G4:  SRel⟶SFun(F,A)⟼(⊓F,P(A)),R:(F,A)⟶(G,B)⟼R[·]:(⊓F,P(A))⟶(⊓G,P(B)),

where ⊓*F*(*C*) = ∩{*F*(*c*)∣*c* ∈ *C*}. Then (*F*_4_, *G*_4_) is an adjoint situation.

**Proof**

Firstly, we show that *G*_4_ is well defined. Let *R* : (*F*, *A*)→(*G*, *B*) be a** SRel**-morphism. For *C*⊆*A*, *b* ∈ *R*[*C*], where *R*[*C*] is defined like Proposition 39, there exists *c* ∈ *C* such that *c* *Rb*. Since *F*(*c*)⊆*G*(*b*) for every *c* ∈ *A*, *b* ∈ *B*, we have ⊓*F*(*C*)⊆⊓*G*(*R*[*C*]), which implies that *R*[·]:(⊓*F*, *P*(*A*))→(⊓*G*, *P*(*B*)) is a** SFun**-morphism. That is, the definition of *G*_4_ is reasonable. Secondly, we claim that (*F*_4_, *G*_4_) is an adjoint situation. It remains to show that **S** **R** **e** **l**((*F*, *A*), (*G*, *B*)) ≈ **S** **F** **u** **n**((*F*, *A*), (⊓*G*, *P*(*B*))). In fact, if *R* : (*F*, *A*)→(*G*, *B*) is a** SRel**-morphism, then *F*(*a*)⊆*G*(*b*) for all *a* ∈ *A* and *b* ∈ *B*, which implies that *F*(*a*)⊆⊓*G*(*R*[{*a*}]). That is, *R*[{·}]:(*F*, *A*)→(⊓*G*, *P*(*B*)) is a** SFun**-morphism. Consequently, assume that *f* : (*F*, *A*)→(⊓*G*, *P*(*B*)) is a** SFun**-morphism. Define a relation *R* : *A* → *B* by *a* *Rb* if *b* ∈ *f*(*a*). Then *a* *Rb* implies *F*(*a*)⊆⊓*G*(*f*(*a*)), whence *F*(*a*)⊆*G*(*b*) and *R* : (*F*, *A*)→(*G*, *B*) is a** SRel**-morphism.

**Definition 49 .**

Assume that *P*, *I* : **S** **R** **e** **l** → **S** **R** **e** **l** are defined by *P* = *F*_3_∘*G*_1_ and *I* = *G*_3_∘*G*_1_. Then *P* and *I* are called the projective cover and injective hull functor, respectively.

**Proposition 50 .**

Consider

(36) $$\begin{array}{c} {\;}^{\prime }\circ P=I\circ {\;}^{\prime }. \end{array}$$

**Proof**

It is straightforward from Theorems 42 and 44.

## 6. Conclusions

Soft set theory, a new powerful mathematical tool for dealing with uncertain problems, has recently received wide attention in both the real-life applications and the theory studies. In recent years, the combination of soft set theory and category theory has resulted in many interesting research topics. In this paper, we mostly focus on offering theoretical results by combining soft set theory and category theory. In other words, we have provided a categorical viewpoint for soft set theory and the results given in this paper can further enrich soft set theories. Particularly, we have proved that the category** SFun** is Cartesian closed, which will provide an important theoretical background for theoretical computer sciences. Naturally, applying our results to other fields such as information sciences and logic is also a valuable work and we will present it in the future work.
